# Depression, Non-Suicidal Self-Injury, and Suicidality in Adolescents: Common and Distinct Precursors, Correlates, and Outcomes

**DOI:** 10.20900/jpbs.20210018

**Published:** 2021-10-14

**Authors:** Zeynep Başgöze, Andrea Wiglesworth, Katherine A. Carosella, Bonnie Klimes-Dougan, Kathryn R. Cullen

**Affiliations:** 1 Department of Psychiatry and Behavioral Sciences, School of Medicine, University of Minnesota, Minneapolis, MN 55454, USA; 2 Department of Psychology, University of Minnesota, Minneapolis, MN 55455, USA

**Keywords:** depression, non-suicidal self injury, suicidal thoughts and behaviors

## Abstract

Depression, non-suicidal self-injury (NSSI), and suicidal thoughts and behaviors (STB) often emerge during adolescence. Despite considerable overlap in clinical presentation, risk factors, and implicated neurobiology, there is also evidence for divergence in terms of precursors, correlates, and outcomes. The complex interrelationships amongst these three clinical domains require considering both shared and divergent patterns of risk for depression, NSSI, and STB; a clearer understanding of these developmental trajectories will be needed to guide optimization and tailoring of early interventions.

Depression, non-suicidal self-injury (NSSI), and suicidal thoughts and behaviors (STB) are serious clinical problems that frequently emerge during adolescence, a time notable for significant brain development. Given that each of these clinical problems can independently and conjointly predict future self-harm and death by suicide [1-5], there is an urgent need to better understand and appropriately address each one. Studying these clinical phenomena can be challenging because while they sometimes manifest independently [6-8], they also frequently co-occur in adolescence [3,4,9-11], making them difficult, if not impossible, to disentangle. Here we offer a perspective on how to conceptualize and navigate these challenges.

First, given the importance of early identification and intervention for improving outcomes, a priority of the field is to understand the premorbid risk factors contributing to the emergence of depression, NSSI, and STB in adolescence. Studies on genetic [12-14], psychosocial, and clinical risk factors (e.g., family functioning, attachment, peer relationships, anxiety, hopelessness [15-17]) have demonstrated both overlap and distinction in their predictive ability for these outcomes. Indeed, NSSI and depression might emerge together as a result of common genetic, cognitive, or social risk factors [18], such as being born to and raised by parents with depression, experiencing trauma or abuse in the home or within the peer context, history of STB, earlier sexual activity, and identifying as a member of a sexual minority [16,19]. It is crucial to understand how multiple risk factors converge to enhance risk. For example, genetic risk factors interact with adverse experiences (e.g., trauma like maltreatment, stressor like poverty) to shape early brain development, especially during critical periods [20], which may set the stage for risk of depression, NSSI and STB [21].

Second, depression, NSSI, and STB should be considered in the context of multiple timescales. Perhaps the most important timescales are the developmental phases across childhood and adolescence, and the evolution of risk states over time (e.g., illness progression). With respect to development, evidence suggests that while depression, NSSI, and STB commonly overlap in adolescents, this overlap is much less present in children. For example, data from the Adolescent Brain and Cognitive Development (ABCD) study suggest that in late childhood, a diagnosis of depression and the occurrence of suicidal thinking are almost mutually exclusive [22]. Indeed, while adolescents who died by suicide commonly had depression, these children are more likely to have had attention deficit hyperactivity disorder [23]. This underscores the importance of placing findings about depression, NSSI and STB into the context of development. With respect to illness progression, although most studies on adolescents who engage in NSSI have reported high rates of depressive symptoms, the directionality of this relationship is unclear. On one hand, since NSSI is often used as an emotion regulation strategy for relieving emotional pain [24], some adolescents may experience depression first and subsequently develop NSSI as a maladaptive strategy to manage their symptoms. On the other hand, adolescents who first develop NSSI may subsequently develop depressive symptoms as a result of the psychological, interpersonal and/or biological sequelae of repeated NSSI. A third possible trajectory is that depression and NSSI pave the way for later STB [25]. While some data supports this idea [26], others suggest that NSSI and STB emerge in parallel [27]. In adolescents, NSSI is a stronger predictor of future suicide attempts than previous suicide attempts [28] as three fourths of suicide deaths represent the first-ever attempt in this group [29]. This underscores the reality that multiple pathways are possible. Finally, since those with both NSSI and current depression have the greatest suicide risk [30], these risk trajectories may be additive. Advancement is needed in understanding how the developmental sequence of the emergence of depression, NSSI, and STB may vary across individuals, and how this knowledge may inform progression of risk states within individuals.

Third, we have advocated for applying a systems neuroscience approach consistent with the RDoC initiative [31] to methodically examine the neurobiological systems implicated in the clinical problems which place youth at risk for suicide. For instance, one of the most studied neurobiological systems to date in adolescent depression, NSSI, and STB is the threat system, which involves the hypothalamic-pituitary-adrenal (HPA) axis [32,33], amygdala [34], and medial prefrontal cortex (mPFC) [35]. One study showed that heightened cortisol reactivity to social stressors predicted later suicidal ideation [36], and our group found that adolescents with depression without NSSI show heightened salivary cortisol in response to a stressor while those with both depression and NSSI demonstrate a flattened cortisol response [37]. Similarly, in another sample, we showed that among adolescents with NSSI, cortisol flattening was most prominent in those with severe NSSI [38]. This flattened pattern was most pronounced for those who engaged in NSSI with a history of suicide attempts [37,38], suggesting that this pattern may become more accentuated along the course of progression of suicide risk, at least among those with NSSI. Studies examining threat system neural circuitry have demonstrated reduced gray matter volume of the anterior cingulate cortex, a key emotion regulatory region, both in youth with STB [39] and with NSSI [40]. Alterations in amygdala-frontal connectivity have been documented in youth with NSSI [38,41] and with high suicide risk [42]. Furthermore, lower amygdala-mPFC connectivity was most pronounced in adolescents with NSSI who also a history of suicide attempt [38], again suggesting a biological pattern that may evolve as risk suicide risk increases.

Together, these examples highlight the need for longitudinal studies that incorporate careful approaches to disentangle overlapping neurodevelopmental trajectories underlying the evolving risk for depression, NSSI, and STB across childhood and adolescence, a task impossible in the context of cross-sectional studies. While some approaches have compared adolescents with NSSI or STB versus healthy controls, this raises the concern that the findings may be largely driven by underlying comorbidity. On the other hand, given evidence that clinically-relevant NSSI is associated with high levels of psychiatric comorbidity [43], approaches seeking to study NSSI in the absence of comorbid psychiatric disorders risk losing external validity. Other approaches include examining NSSI and/or STB within the context of depression (e.g., comparing adolescents with depression with versus without NSSI or STB), or a take-all-comers approach in which both risk and “control” groups include multiple types of psychopathology, and in either case, including statistical controls for underlying comorbidity. Each of these approaches has its strengths and weaknesses. Moving forward, additional method development is needed for clearly delineating developmental trajectories. One promising approach may be the use of person-centered quantitative analyses such as latent class analysis, which allow examining multiple related factors simultaneously within the individual over time [44]. In any case, appropriately powered, longitudinal research will be required to delineate the temporal course of how each clinical phenomenon and its neural signature unfolds in relation to the others over time. Advancing this knowledge is required to guide precision of assessments and early intervention to reduce death by suicide and improve outcomes for at-risk youth.
